# Numerical Study for Efficient Cooling of Perishable Food Products During Storage: The Case of Tomatoes

**DOI:** 10.3390/foods14142508

**Published:** 2025-07-17

**Authors:** Audrey Demafo, Abebe Geletu, Pu Li

**Affiliations:** 1 Department of Physics, College of Sciences and Technology, University of Rwanda, Kigali P.O. Box 4285, Rwanda; audrey.demafo@aims.ac.rw; 2 German Research Chair, Applied Mathematics and AI, African Institute of Mathematical Sciences (AIMS), KN 3 Rd, Kigali P.O. Box 7150, Rwanda; abebe.geletu@aims.ac.rw; 3 Group of Process Optimization, Institute for Automation and Systems Engineering, Technical University of Ilmenau, P.O. Box 100565, 98684 Ilmenau, Germany

**Keywords:** perishable crops, cooling, airflow pattern, heat transfer, package design

## Abstract

Unveiling temperature patterns within agricultural products remains the most important indicator for their quality assessment during post-harvest treatments. Temperature control and monitoring within vented packages is essential for preserving the quality of perishable goods, such as tomato fruits, by preventing localized temperature maxima that can accelerate spoilage. This study proposes a modeling and simulation approach to systematically investigate how ventilation design choices influence internal airflow distribution and the resulting cooling performance. Our analysis compares three distinct venting configurations (single top vent, single middle vent, and two vents) across two package boundary conditions: an open-top system allowing for dual air exits through the open top boundary and the outlet vent(s), respectively, and a closed-top system with a single exit pathway through the outlet vent(s). All scenarios are simulated to assess airflow patterns, velocity magnitudes, and temperature uniformity within different package designs.

## 1. Introduction

Temperature is regarded as the most important environmental factor affecting the quality of fresh agricultural products and their shelf life during post-harvest treatments (e.g., pre-cooling, storage, transportation, etc.) [1,2]. Pre-cooling the products right after the harvest is the first step to proceed with in order to prevent their rapid deterioration [3]. In fact, climacteric crops such as tomatoes continue their metabolism even after their separation with the parent plant; as a result, some biological reactions (e.g., respiration and transpiration) continue to take place, thereby inducing internal heat generation within the products with irreversible consequences on their quality [4]. Therefore, it is of great necessity to cool the products as fast as possible to slow down the reaction rate of the aforementioned biological processes. During the cooling process, maintaining temperature homogeneity and optimal airflow circulation within food containers is essential to ensure the long-term quality preservation of the products [5]. Package configurations, product size and geometry, product arrangement, and the thermophysical properties of products, just to name a few, are among the most influential criteria to ensure an optimal cooling performance. In recent years, interest in optimizing packaging systems has significantly increased given their importance. Not only are food packages important in the cooling process, but they also serve as a protection of the products against environmental damages.

**Literature review on the impact of packaging configurations on product cooling performance**

A plethora of insightful studies have been published in the past, proposing various package designs for the cooling of different types of agricultural products.

For instance, the work in [6] investigated how different vent setups in packages affect the cooling and airflow around fresh products when using forced-air cooling. Packages with 1, 3, and 5 vents were tested for their contribution to the cooling of the products. Three different vents configurations were simulated and validated experimentally with an inlet airflow rate of 0.022 m^3^.s^−1^. It was concluded that increasing the number of vents from 1 to 5 resulted in more uniform cooling. In an earlier study, the same authors in [7] studied the simultaneous effect of airflow and temperature when cooling stacked spheres inside ventilated packages. The study focused on how different vents sizes affect the airflow distribution and cooling uniformity. Results indicated that increasing vent size significantly improves airflow uniformity and reduces cooling heterogeneity. Their findings highlight the critical role of vent design in optimizing product cooling to ensure quality during post-harvest storage and transport.

In [8], an integrated approach combining experimental research and computational fluid dynamics (CFD) modeling was proposed to evaluate the cooling performance of ten apple cartons widely used in China’s agricultural cold-chain logistics. The study aimed to optimize forced convection cooling (FAC) processes by analyzing airflow and heat transfer within various packaging designs, focusing on energy consumption, cooling rate, cooling uniformity, and fruit quality parameters such as chilling and mass loss. Their results showed that symmetrical and homogeneous placement of vents significantly improves the FAC efficiency, by promoting uniform cooling and temperature distribution within the carton. In contrast, it was found that increasing the vent area or changing the vent shape with regard to its symmetric positioning does not guarantee improved cooling efficiency.

A three-dimensional model was developed in [9] to predict airflow and heat transfer in a pallet layer of tomatoes. The model was validated by experimental results and was subsequently used to optimize temperature homogeneity by adjusting airflow rate and crate design. The study evaluated the influence of three crate designs on the cooling kinetics and temperature uniformity during FAC. A similar study can be found in [10], where different vent holes designs were simulated to evaluate their effect on airflow and temperature distribution.

The authors in [11] studied how the design of secondary packaging trays affects the pre-cooling efficiency and temperature uniformity of strawberries packaged in airtight clamshells (AC) by simulating modified atmosphere packaging (MAP). Using laboratory experiments with artificial strawberries, the study compared one existing commercial tray design to three new alternatives, focusing on the influence of air head space, vent hole area, and inlet airflow rate on the cooling performance.

Coorey et al. [12] critically reviewed how the cooling rates of ready-to-eat food products are influenced by the type of food containers used. A thorough review of the studies that have analyzed the effect of package design on airflow and product cooling is reported in [13]. Similar studies on the impact of packaging designs and their influence on product cooling can be found in [10,14,15,16,17] and the references therein.

**Computational Fluid Dynamics**

The aforementioned and previous other studies on product cooling relied on experimental measurements [18,19], numerical simulations [20,21,22], or a combination of both approaches [6,7,9,23]. Mathematical modeling and numerical simulations are a cost-effective way to gain insight about physical systems without the need for experimental setups, which are usually expensive to realize. However, experimental observations are usually needed to validate the results of the mathematical model. Therefore, instead of conducting an experimental study with many trials and errors until the expected results are found, numerical analysis helps in reducing the occurrence of failure in the experiment, thereby minimizing the loss of experiment time and material resources. To date, the numerical study of heat and mass transport in food packaging during cooling has mainly depended on computational fluid dynamics (CFD). There are two modeling approaches used in the CFD framework, namely direct computational fluid dynamics simulations (DCFDS) and the porous media assumption (PMA) [1,24].

In DCFDS, the geometry of the container is explicitly designed with the help of computer-aided design (CAD) software (e.g., SolidWorks, ANSYS, FreeCAD, etc.). In order to be able to solve the partial differential equations (PDEs) numerically, the entire domain is partitioned into smaller pieces with an appropriate meshing method (finite element or finite volume). The DCFDS approach has demonstrated its power in terms of high accuracy of the obtained results [1,24]. However, the computational time can be extremely high, especially with complex domains such as food containers. In addition, it requires a very good knowledge of the CAD software, with most of them being commercially licensed and therefore not affordable to everyone. To overcome these shortcomings, the porous media assumption is often used. This approach simplifies the complex domain into a continuum where there is no longer discontinuities (e.g., the difference between the air domain and the product domain observed in tomatoes containers). In this way, the domain can be easily designed and the governing equations solved, leading to a low computational cost compared to the DCFDS method. Studies using the PMA approach with application to product cooling can be found in [25,26,27,28,29] and the references therein. Nonetheless, the simplification of the geometry makes the PMA approach less accurate.

**Our contribution**

**Figure 1:**
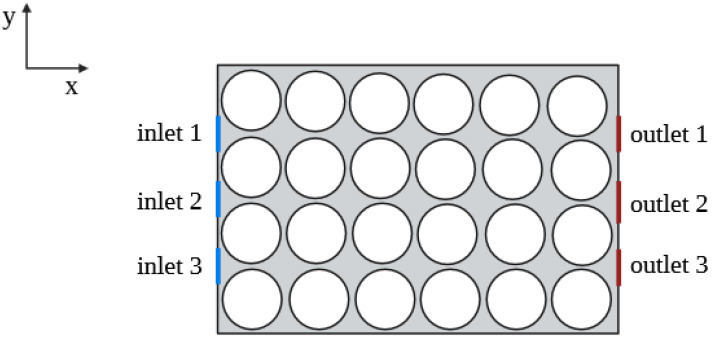
Computational domain with inlet and outlet vents. The inlet vents are located on the left and the outlet vents on the right of the package.

This study presents a modeling and simulation approach to evaluate the temperature distribution influenced by packaging design during the forced-air cooling process, using tomatoes as a case study. Specifically, we explore various ventilation configurations using the CFD-DCFDS method to analyze airflow patterns and temperature distribution within two primary packaging designs: (i) an open-top boundary and (ii) an insulated-top boundary. While previous studies have significantly advanced our understanding of cooling dynamics, the effect of the top boundary remains underexplored. To address this knowledge gap, our study consists of evaluating the cooling performance of open-top and insulated-top cases, combined with lateral venting, with the aim of identifying the most effective design for uniform cooling and optimal airflow distribution. Three venting scenarios are examined: one inlet–one outlet (inlet 1; outlet 3), one inlet–one outlet (inlet 2; outlet 2), and two inlets–two outlets (inlets 1, 3; outlets 1, 3) (see Figure 1 and Table 1).

The remainder of the paper is structured as follows: Section 2 describes the modeling and numerical procedures; Section 3 presents the simulation results; and Section 5 concludes the study.

## 2. Methodology

### 2.1. Geometry Description

We consider a two-dimensional model of a food package as shown in Figure 1. The geometry comprises package dimension of 0.36 m × 0.24 m. The package has rectangular vents located at different positions on its left and right lateral sides, respectively. The inlet vents are located on the left, while the outlet vents are on the right side of the package. All vents have a length of 0.02 m. Circles are chosen as an approximate geometry to represent tomatoes. This is because a vast majority of fruits and vegetables have a nearly spherical shape, which are represented as circles in 2D models. The circles are 0.026 m in radius (0.052 m in diameter) and spaced 7.5 mm apart. The distance between the walls (lateral sides) and the circles is 3.8 mm.

### 2.2. Governing Equations

#### 2.2.1. Airflow Model

Fluid motion in a given domain is dictated by conservation laws of mass and momentum given as follows:

(A) Conservation of mass (*continuity equation* [30])

(1) $$\frac{\partial \rho }{\partial t}+div\left(\rho u\right)=0,$$

where ρ (kg.m^−3^) is the fluid density and u (m.s^−1^) represents the fluid velocity vector. In this study, buoyancy forces are neglected, and the only force driving the airflow is convection. Therefore, air density is assumed to be invariant in time and space. By taking into account this assumption, Equation (1) reads

(2) $$\frac{\partial u_{x}}{\partial x}+\frac{\partial u_{y}}{\partial y}=0,$$

where $u_{x}$, $u_{y}$ represent the components of the flow velocity in the x− and y− directions of the Cartesian coordinate system.

(B) Conservation of momentum (*Navier-Stockes equation* [30])

(3) $$\rho_{a}\frac{\partial u}{\partial t}+\rho_{a}\left(u\cdot \nabla u\right)=-\nabla p+\nabla \cdot \left[\mu_{a}\left(\nabla u\right)+{\left(\nabla u\right)}^{T}\right],$$

where $\mu_{a}$ (Pa.s^−1^) represents the dynamic viscosity of air and $\nabla p={\left(\frac{\partial p}{\partial x}\;\frac{\partial p}{\partial y}\right)}^{T}$ denotes the pressure gradient vector.

The accuracy of the Navier–Stockes equation together with the continuity equation to model airflow patterns in loaded food packages during FAC was validated by [7,31,32] on a similar geometry with a nearly similar product diameter. Their CFD results and experimental observations were generally in good agreement. For instance, in [7], a mean absolute error of only 2.2 °C was reported across all vent configurations. Additionally, their regression analysis comparing predicted with experimental temperatures showed a slope of 1.02 and an intercept of 0.33, with an R^2^ of 0.92, indicating a strong correlation and a minimal bias.

#### 2.2.2. Energy Conservation

The energy conservations in the air and product zones are given separately as follows:

(4) $$\begin{array}{c} \rho_{a}c_{p,a}\frac{\partial T_{a}}{\partial t}+\rho_{a}c_{p,a}\left(u\cdot \nabla T_{a}\right)=\nabla \cdot \left(k_{a}\Delta T_{a}\right), \end{array}$$

(5) $$\begin{array}{c} \rho_{p}c_{p,p}\frac{\partial T_{p}}{\partial t}=\nabla \cdot \left(k_{p}\Delta T_{p}\right), \end{array}$$

where $T_{a}$(K) and $T_{p}$(K) represent the air and product temperature at different positions inside the food package; $\rho_{a}$ and $\rho_{p}$ (kg.m^−3^) denote the density of the air and product; $c_{p,a}$ and $c_{p,p}$ (J.kg^−1^K^−1^) are the specific heat capacity of air and product; and $k_{a}$ and $k_{p}$ (W.m^−1^K^−1^) are the thermal conductivity of the air and product, respectively. The thermophysical properties of air and product domains are assumed constant. The thermophysical properties of tomatoes are assumed to be similar to those of carrageenan gel (see Table 2) due to its high water content, which makes its thermal properties close to those of real tomatoes [9]. In addition, the respiration heat is not taken into account since the forced convection dominates natural convection during the cooling process. Furthermore, the heat generated by respiration is omitted since the cooling process significantly slows down microorganisms’ activity, thereby reducing the respiration rate.

### 2.3. Boundary Conditions

The boundary conditions used in this study are airflow inlet as well as outlet, open boundary, thermal insulation, and the no-slip boundary condition on the package walls. At the inlet vent(s) of each model configuration, a constant airflow velocity of u=0.25 m.s^−1^ and inlet temperature of 4 °C are applied. The choice of this inlet temperature is motivated by previous experimental studies that considered the same temperature range for product cooling [33,34]. In addition, it is reported that a low temperature below 8 °C enhances pre-cooling and effectively prevents earlier spoilage [3].

An outlet pressure $p_{0}$ = 0 Pa is imposed at the outlet vent(s). The pressure in the software are calculated relative to the atmospheric pressure. This implies that a positive pressure (p>0) is above the atmospheric pressure, while a negative pressure (p<0) is below it. A pressure of 0 Pa corresponds to the atmospheric pressure assumed as 1 atm.

Two boundary conditions are considered at the top of the package to study and compare their effect on the cooling rate and uniformity. In the first scenario, an open boundary is considered, and a “no viscous stress” condition applied. This implies that the air is free to flow in and out of the domain without any constraints [35]. In addition, a convective heat transfer condition is applied at the open boundary to allow heat transfer between the product and the ambient air. In the second case, the top boundary is assumed to be insulated, i.e., the package is covered. In addition, the walls of the package are assumed to be thermally insulated. Furthermore, a no-slip condition, $u_{walls}=0$ m.s^−1^, is imposed on the walls to prevent airflow through them.

### 2.4. Numerical Procedure

We solve the above defined problem by using the finite element analysis feature available in the CFD software tool Comsol Multiphysics (Comsol Inc., Burlington, MA, USA), version 6.2 [36]. The velocity field is approximated using quadratic Lagrange elements, while the pressure and temperature fields are respectively approximated with linear Lagrange elements, on a triangular mesh. A sensitivity analysis is conducted to obtain an optimal mesh that can reflect the process dynamics as accurately as possible. For this purpose, several mesh densities are used and tested on the geometry, with the governing equations of the airflow.

In Comsol Multiphysics, mesh density levels include various options: *extremely coarse, extra coarse, coarser, coarse, normal, fine, finer, extra fine*, and *extremely fine* [36]. Given the importance of an accurate representation of airflow pattern and temperature distribution within the package, three levels of fine mesh are investigated. The resulting mesh statistics in all three configurations are reported in Table 3. The mesh quality is evaluated based on the skewness parameter, which measures individual elements’ distortion in the mesh. A minimum element quality of 0.25 is commonly used as the threshold for assessing whether a mesh meets the acceptable quality standard [10]. Dehghannya et al. [7] reported that an average mesh quality greater that 0.3 is unlikely to affect the accuracy of the numerical solution. In order to further enhance mesh quality, contact between individual products and between a product and the boundaries is avoided. The products are spaced 7.5 mm apart while the distance between the boundaries and the products is set to 3.8 mm. Mesh refinement is applied near the vents in order to accurately capture the relevant dynamics at these zones.

The accuracy of the numerical solutions is assessed based on the residual errors for continuity, momentum, and energy equations. In addition, the selection of the optimal mesh density relies on the convergence criterion of the solver. A fully coupled linear iterative solver (GMRES) is used for solving the fluid flow problem, and a direct linear solver (PARDISO) for the energy conservation equations. The continuity and momentum equations are solved in a steady-state mode, and the obtained results are used as inputs to solve the energy equations. The total solution time for the simulations was 4 h 40 min on a Core i7 PC, under the Windows OS. Simulations of the transient problem are performed over a period of 1800 min (30 h).

**Figure 2:**
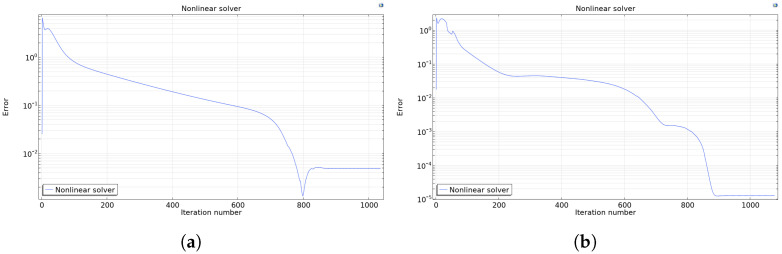
Error magnitude of the nonlinear solver for finer (**a**) and extremely fine (**b**) meshing scenarios.

After several testing runs, an extremely fine mesh was selected as it leads to the lowest error, approaching the magnitude of $10^{-5}$, while the error magnitude in the *fine* and *finer mesh* cases was of the order of $10^{-3}$ (see Figure 2).

### 2.5. Performance Metrics

Time parameters are the most reported metrics used to measure the performance of a product cooling process [37]. In this regard, the half cooling time (HCT) and the seven-eighths cooling time (SECT) are employed, and the corresponding system states evaluated.

The half cooling time (HCT) corresponds to the time when the temperature difference between the products and the cooling temperature reaches half of its initial value:

(6) $$\Delta T\left(t_{1/2}\right)=\frac{1}{2}\left(T_{p,init}-T_{cool}\right).$$

The seven-eighths cooling time (SECT) corresponds to the time when the temperature difference between the products and the cooling temperature reaches $1/8^{th}$ of its initial value:

(7) $$\Delta T\left(t_{7/8}\right)=\frac{1}{8}\left(T_{p,init}-T_{cool}\right).$$

In Equations (6) and (7), $T_{p,init}$ and $T_{cool}$ represent the initial temperature of the products and the ambient cooling temperature, respectively.

## 3. Results and Discussion

### 3.1. Airflow Pattern

**Figure 3:**
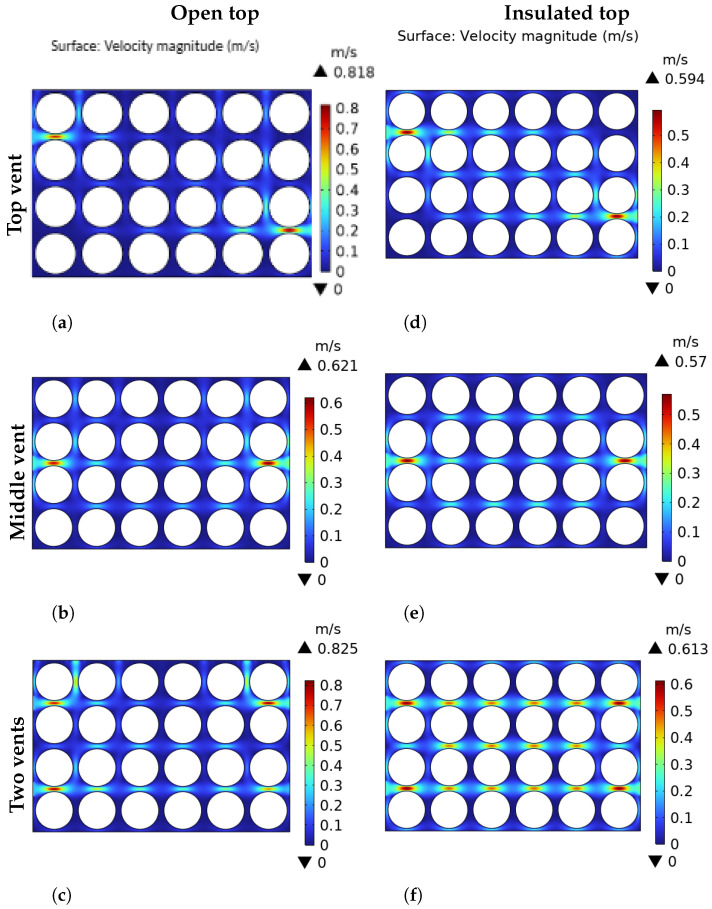
Velocity magnitude comparison across different venting scenarios in open top boundary (**a**–**c**) and closed top boundary (**d**–**f**), respectively.

Figure 3 shows the airflow pattern from inlet to outlet vents through the array of products for different package configurations. The results for the open-top (Figure 3a–c) and the insulated-top (Figure 3d–f) scenarios are presented. For the top vent configuration (inlet: pos. 1; outlet: pos. 3’) under the open top scenario (Figure 3a), it is observed that the air flows into the package through the vent and immediately exits through the path of least resistance—the open top. In contrast, in the insulated scenario (Figure 3d), the closed boundary forces the air to travel further across the package towards the outlet vent (pos. 3’). This results in slightly better penetration into the central rows compared to the open top case where a large dead zone (dark blue zones) is observed in the central region of the array.

For the middle vent in the open-top package (Figure 3b), the air entering from the inlet vent is pulled upwards to the low-pressure open boundary at the top. This results in an asymmetric flow path, with better ventilation in the upper-middle section and poor ventilation at the bottom. When the middle vent is made on the package with an insulated top (Figure 3e), a more symmetric flow path is observed, and the dead-zone areas are significantly reduced. The two-vent configuration under the insulated-top scenario appears to be the most effective design for the airflow pattern. In Figure 3c, the air entering from the top vent directly escapes the domain through the open top in a similar way to what was previously described. Although the overall flow rate is high, the airflow is not distributed in an effective manner throughout the package. While the insulated top package presents an overall lower velocity compared to the open-top case, the closed boundary promotes a uniform air distribution throughout the entire package (Figure 3f). These results demonstrate the efficacy of the proposed mathematical model as a fairly satisfactory description of the practical situation. It is worthwhile to note that the model has been validated through experiments conducted in [6,7,32].

### 3.2. Temperature Distribution

**Figure 4:**
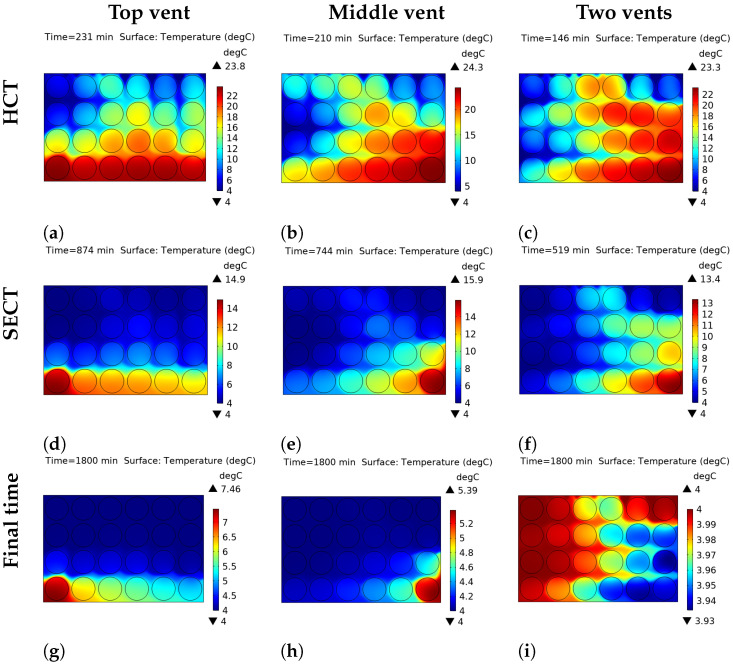
Thermal heterogeneity at half cooling time (**a**–**c**), seven-eighths cooling time (**d**–**f**), and final time (**g**–**i**) for the open-top package with different vent locations.

**Figure 5:**
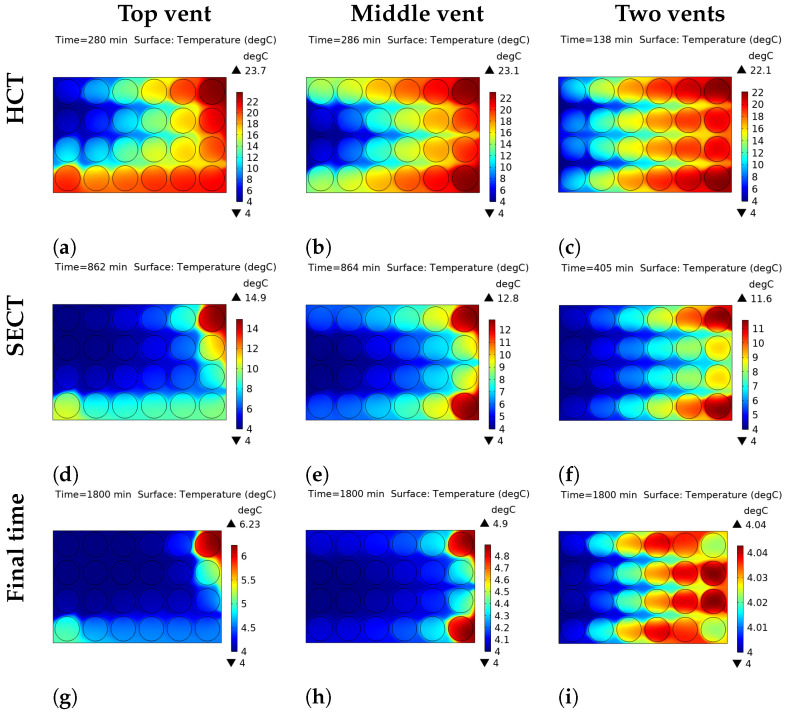
Thermal heterogeneity at half cooling time (**a**–**c**), seven-eighths cooling time (**d**–**f**), and final time (**g**–**i**) for the insulated-top package with different vent locations.

**Figure 6:**
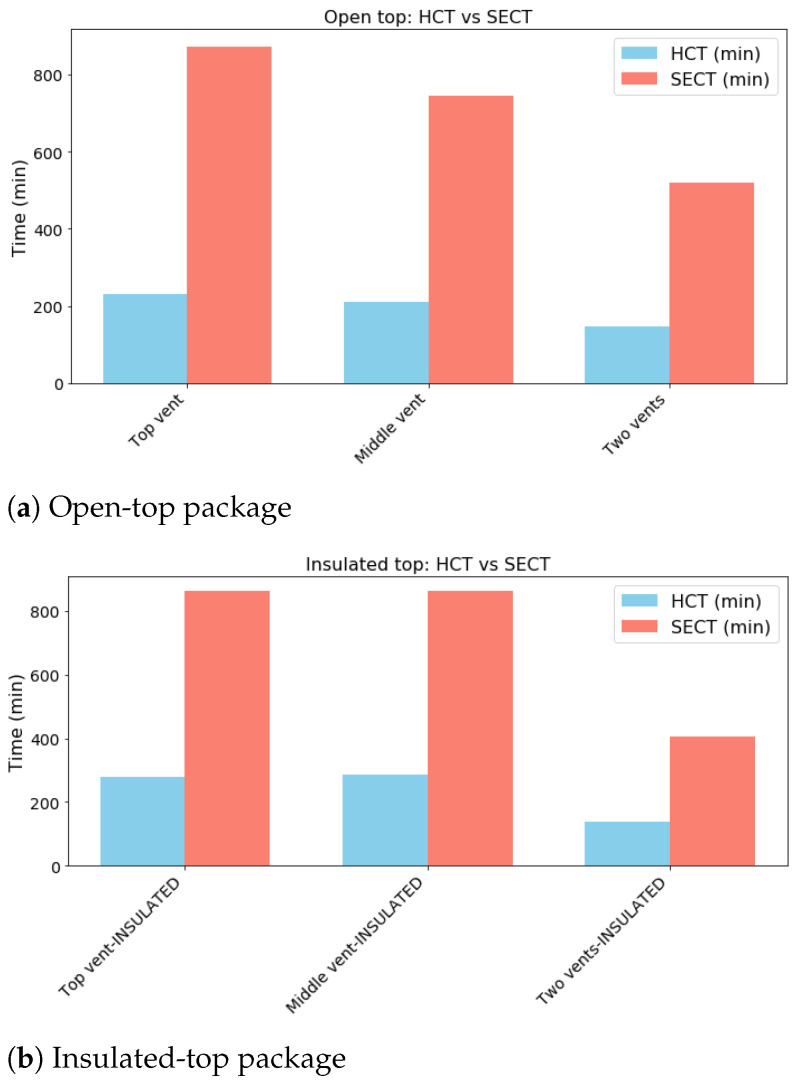
Half cooling time and seven-eighths cooling time for all package configurations.

The thermal heterogeneity within the open-top package and the insulated-top package, under all vent configurations is presented in Figure 4 and Figure 5, respectively. The performance of the open-top package, where convective heat transfer is permitted at the top boundary, is significantly different from that of the insulated top package. The results are given at half cooling time (HCT), seven-eighths cooling time (SECT), and the final cooling time, for all the venting configurations. The average temperature within the package is used to evaluate the temperature difference corresponding to the HCT and the SECT, respectively. HCT and SECT values for the open-top and the insulated-top scenarios are reported in Table 4, and the corresponding graphical illustration is shown in Figure 6.

#### 3.2.1. Effect of Vent Configuration on the Cooling Performance

A comparative analysis of the vent configurations reveals that the two-vent system provides substantially more rapid and uniform cooling than either the top or middle single-vent configurations across both top boundary conditions. For the open-top package, the HCT is achieved after 146 min with two vents (Figure 4c), leading to a significant time reduction compared to 210 and 231 min for the single-vent configurations (Figure 4a,b). This disparity in performance is further illustrated at the SECT stage. With the single-vent configuration, significant thermal gradients persist (Figure 4d,e), whereas the two-vent system exhibits a slightly higher degree of uniformity (Figure 4f). At the final cooling time point, the two-vent configuration achieves the best temperature uniformity. Using the insulated top package, the final temperature difference ($T_{max}-T_{min}$) across the package is only 0.04 °C with two vents (Figure 5i), whereas it remains as high as 2.23 °C under the top-vent configuration and 0.9 °C under the middle vent configuration (Figure 5g,h).

#### 3.2.2. Influence of the Top Boundary Condition

The top boundary condition—convective (open) versus adiabatic (insulated)—was found to be a critical determinant of both cooling rate and thermal uniformity. It is found that, for the open-top configuration that permits convective heat transfer from the top surface, the cooling process is generally accelerated, especially in single-vent cases. The influence of the top boundary is also visible at the SECT stage. Notably, for the two-vent system, the insulated-top package reached the SECT faster (405 min, Figure 5f) than the open-top package (519 min, Figure 5f), highlighting the efficiency of the single cooling pathway in this configuration. However, a key finding is observed when comparing the final uniformity in the two-vent configuration. The insulated top package yielded a more uniform final temperature within the package. As shown in Figure 4 and Figure 5i, the final temperature difference was reduced from 0.07 °C in the open-top model to 0.04 °C in the insulated-top model, indicating a higher degree of thermal uniformity in the latter scenario.

## 4. Discussion on Thermal Heterogeneity Results

The results highlight a crucial relationship between forced air convection through the side vents and the nature of the top thermal boundary. The superior performance of the two-vent system can be attributed to an efficient airflow rate. This contributes to reduction of the cooling time and prevents the formation of stagnant hot spots. The SECT plots are particularly informative, as they demonstrate the inefficiency of single-vent systems to effectively remove heat from the package during slower stages of cooling.

Results regarding the influence of the top boundary condition were particularly insightful. The open-top configuration has a dual cooling mechanism, where heat is removed simultaneously via the side vents and the convective top surface. This additional heat transfer pathway at the top surface explains the fast cooling observed, especially in the single-vent cases. However, it also introduces a competing top-down cooling pathway, which creates a vertical temperature gradient and results in slightly lower final uniformity.

In contrast, the insulated-top configuration reduces the cooling mechanism to a single pathway through the side vents. By preventing top-down cooling, the vertical temperature gradient is avoided, and only a horizontal cooling front is allowed. This single-pathway cooling mechanism is the reason behind the superior final temperature uniformity observed in Figure 5g–i.

## 5. Conclusions

This study investigates the airflow pathway and temperature distribution within two main package designs with different venting configurations (“top vent”, “middle vent”, “two vents”). We consider a package with an open-top boundary and a package with the top boundary insulated. A modeling and simulation approach within the CFD framework is used to setup the underlying governing equations of the problem, which are numerically solved. The cooling rate and cooling uniformity of the two package designs are evaluated via the half cooling time (HCT) and the seven-eighths cooling time (SECT).

Regarding the airflow inside the package, it is observed that the two-vent system offers the most effective flow rate, especially in the insulated-top case. The results show that the free open boundary obstructs the airflow circulation and generates large dead zones within the package. This can be a serious threat to the product shelf-life, since sufficient air circulation significantly contributes to extend product lifetime.

As far as the temperature distribution is concerned, it is shown that vent positions and the top thermal boundary condition significantly influence the cooling uniformity in both design scenarios. Using the open-top package, the HCT is shorter (146 min), compared to 210 and 231 min for the single-vent systems. At the SECT stage, the thermal gradient inside the package is smaller in the two-vent package compared with the single-vent configurations. It is further shown that the insulated-top package design promotes a better cooling uniformity across the package due to the single-pathway cooling mechanism. Allowing heat transfer from the top surface by the open top design introduces a top-down cooling pathway, which generates a vertical temperature gradient and results in a slightly lower uniformity.

As a result, for applications like food cooling, where ensuring every product item receives adequate ventilation to prevent spoilage is critical, the insulated-top package with two vents is the better design. It guarantees the most uniform and controlled environment for the entire package.

While the results of this study are promising, further investigations need to be considered: (1) Improvement in reducing the simulation time is needed. Thus far, the DCFDS approach is time consuming and resource demanding, which makes it prohibitive for real-time responses. As mentioned earlier in the numerical procedure section, the maximum simulation time was more than four hours. Improvements can be made to considerably reduce the computational time while preserving the structure of the model. (2) Improvement is also needed on the package geometry. In practice, several packages stacked on top of each other are subject to cooling. It is therefore important to upgrade the current model to a more realistic 3D scenario.

## Figures and Tables

**Table 1:** Vents’ location.

Vent Configuration	Inlet Position	Outlet Position
One inlet–one outlet	1	3’
One inlet–one outlet	2	2’
Two inlets–two outlets	1, 3	1’, 3’

**Table 2:** Thermal properties of carrageenan gel [9].

Properties	Value
Specific heat, $c_{p}$ (J.kg^−1^.K^−1^)	4100
Thermal conductivity, λ (W.m^−1^.K^−1^)	0.519
Density, ρ (kg.m^−3^)	1013

**Table 3:** Mesh statistics.

Mesh Densities	Total No. of Elements	MEQ	AEQ	Element Area Ratio	Mesh Area
Fine	6834	0.221	0.7865	9.571 × $10^{-4}$	0.0864 (m^2^)
Finer	8589	0.1072	0.7982	1.108 × $10^{-3}$	0.0864 (m^2^)
Extremely fine	110,662	0.2975	0.8702	1.161 × $10^{-3}$	0.0864 (m^2^)

**MEQ:** Minimum element quality; **AEQ:** Average element quality.

**Table 4:** Half cooling time and seven-eighths cooling time for all package configurations.

Open top	**Time constants**	**HCT**			**SECT**		
	**Pckg. Conf.**	conf. 1	conf. 2	conf. 3	conf. 1	conf. 2	conf. 3
	**Times (min)**	231	210	146	874	744	519
Insulated top	**Time constants**	**HCT**			**SECT**		
	**Pckg. Conf.**	conf. 1	conf. 2	conf. 3	conf. 1	conf. 2	conf. 3
	**Times (min)**	280	286	138	862	864	405

Conf. 1 is the one inlet–one outlet configuration (inlet: pos. 1; outlet: pos. 3’), conf. 2 is the configuration with the middle vent, and conf. 3 is the two vents configuration.

## Data Availability

The original contributions presented in this study are included in the article. Further inquiries can be directed to the corresponding author.
